# The value of reporting cases

**DOI:** 10.3402/jchimp.v1i4.15884

**Published:** 2012-01-26

**Authors:** Robert P. Ferguson

When I wrote my first case report and it was published in a premiere medical journal (1), I was thrilled. The acceptance meant the world to me. It was a remarkable case with a unique combination of coexisting problems. Most importantly, we thought it was a one-of-a-kind case of cryptococcal infection emerging in a Cushingoid patient who had become adrenally excessive by virtue of an ACTH-secreting lung cancer. We thought it was the first case of disseminated cryptococcosis secondary to endogenous steroid excess. Previously, cryptococcal infections secondary to Cushing's syndrome had been associated with exogenous steroids. Four months later, a letter to the editor in the same journal (2) pointed out that our literature review had missed a previous similar case published in the *New England Journal of Medicine* in 1959 (3). The respondent closed with ‘Does it matter who is first?’

Many years later (and hopefully wiser), I should not have been crushed. Uniqueness is nice in case reports but not essential. What is essential is the case report exercise itself. The process requires examination of details, careful documentation and recording, and communication. It requires an understanding of the appropriate literature. Even if the case is not unique, it may be the exception that proves the rule. What makes a case publishable? It is publishable if, after introducing the reader to what lies ahead, the reader is interested in reading on to learn. The case report should be contributory to the knowledge of readers. It enlightens them and makes them think about their own experiences. In 1930, Fuller Albright and others reported the case of Dr. Charles Martel, a sea captain who later developed osteitis fibrosis cystica from one of the first diagnoses of hyperparathyroidism secondary to a parathyroid adenoma (4). The images depicted in the journal stuck with me when I first saw them in medical school. We recently had a grand rounds presentation at our hospital on hyperparathyroidism from an internationally known expert. He showed the same Martel photos that I vividly remember from my first days in physiology. Case reporting has been at the heart of medical care for generations.

However, peer-reviewed published case reports have become an endangered species. This is not a new development and has been acknowledged for more than 20 years (5). Part of this has to do with the low ‘impact factor’ of case reports. Impact factor is based on the number of times published articles are cited. Journals benefit from articles being widely cited. Case reports are usually not widely cited and hence have a lower impact factor. This may well be, but it still does not diminish the value of case reports. Dan Reisenberg said it all 25 years ago: it is ‘blasphemy’ for journals to refuse to publish case reports (6). He pointed out that the most important factor was the will to do a good case report. In the *Canadian Medical Journal*, Bruce Scriers emphasized that case reports should be offering new insights and that important cases should be presented briefly and clearly (7).

In response to the lack of venue for publishing case reports, the *Journal of Case Reports* appeared in 2007 (8). It publishes case reports exclusively. The journal pointed out that detailed individual information was an important contributor to medical education. At the *Journal of Community Hospital Internal Medicine Perspectives*, we fully agree with this position. These bits and pieces that we accumulate throughout our years in the medical profession expand our fund of medical knowledge. Individual cases understood in depth are superb learning tools leading to ‘I have seen this before.’

Why should residents do case reports? Residents who do case reports experience an elegant form of practice-based learning. Critically reviewing a case report, applying it to your next patient, improving patient care, and improving your medical knowledge are a recipe for professional growth. What makes a good case report? It may be something unique that has not been seen before, or may be the exception that proves the rule. In addition, good case reports interest the reader and improve patient care in general. Case reports contribute to the medical literature. Writing case reports is an important exercise and should lead to writing succinctly and in depth with clarity. From my editor's point of view, having communicated with many residents and students who have submitted case reports over the last year to *JCHIMP*, the individual scholarly growth associated with this classic academic exercise is clearly evident.

In this issue of the journal, we have six manuscripts dealing with case reports. They are summarized with the other articles elsewhere in this issue. My favorite is a report of two cases of meningococcemia. Both of these cases were fatal in healthy adults, and both received autopsies. One revealed the full-blown syndrome known as Waterhouse-Friderichsen syndrome, described in 1911 in the *Lancet* by Waterhouse of three episodes of meningococcemia leading to adrenal hemorrhage and death (9). Our two cases personify why they should be published. They are extremely interesting cases that many readers have never seen and serve to contrast some patients with meningococcemia as opposed to full-blown Addisonian crisis. They lead the authors and readers to review literature and understand not only the history of meningococcemia but also the unique virulence of this aggressive organism going back to Dr. Waterhouse's article published 100 years ago.

*Robert P. Ferguson, MD* Editor
